# Successful Treatment of Persistent Postcholecystectomy Bile Leak Using Percutaneous Cystic Duct Coiling

**DOI:** 10.1155/2015/273198

**Published:** 2015-12-21

**Authors:** Vinay Rai, Akin Beckley, Anna Fabre, Charles F. Bellows

**Affiliations:** ^1^Department of Surgery, University of New Mexico, Albuquerque, NM 87131, USA; ^2^Department of Radiology, University of New Mexico, Albuquerque, NM 87131, USA

## Abstract

Laparoscopic cholecystectomy is one of the most commonly performed operations worldwide. Cystic duct is the most common site of bile leak after cholecystectomy. The treatment of choice is usually conservative. Using sufficient percutaneous drainage of the biloma cavity and endoscopic retrograde cholangiography (ERCP) with sphincterotomy and/or stenting, the cure rate of bile leaks is greater than 90%. In very rare cases, all of these measures remain unsuccessful. We report a technique for the successful treatment of persistent cystic duct leak. After failed ERCP and stenting, bile leak was treated by coiling the cystic duct through a drain tract. This technique is safe and effective and helps avoid the morbidity of reoperation.

## 1. Introduction

Cystic duct leak is the commonest biliary complication of cholecystectomy [1, 2]. Frequency of cystic duct leak ranges from 0.07 to 0.63% in large series [3]. Endoscopy with sphincterotomy and stenting is the first line of treatment with a success rate greater than 90% [2]. If this option fails, reoperation with ligation or reclipping of cystic duct has been described [4]. However, this is associated with high morbidity and mortality.

We present a case report of a patient who underwent urgent subtotal cholecystectomy complicated by the development of a persistent, controlled bile leak. This was successfully managed using a novel technique by coiling the cystic duct through the drain tract. This case demonstrates an alternative option to treat this complication of cholecystectomy and avoid a high-risk operation.

## 2. Case Presentation

A 54-year-old morbidly obese female with COPD, diabetes, hypertension, anxiety, and depression presented with right upper quadrant pain, leukocytosis, and fever. Ultrasound confirmed acute cholecystitis. Bile duct was of normal size and liver function tests were normal.

Patient was taken to the operating room for an urgent laparoscopic cholecystectomy. However, upon visualization, gallbladder was gangrenous and conversion to an open subtotal cholecystectomy became necessary due to inability to safely dissect inflammatory adhesions and failure to clearly delineate the anatomy. The gallbladder remnant was closed and a Jackson-Pratt drain was placed in the gallbladder fossa.

**Figure 1:**
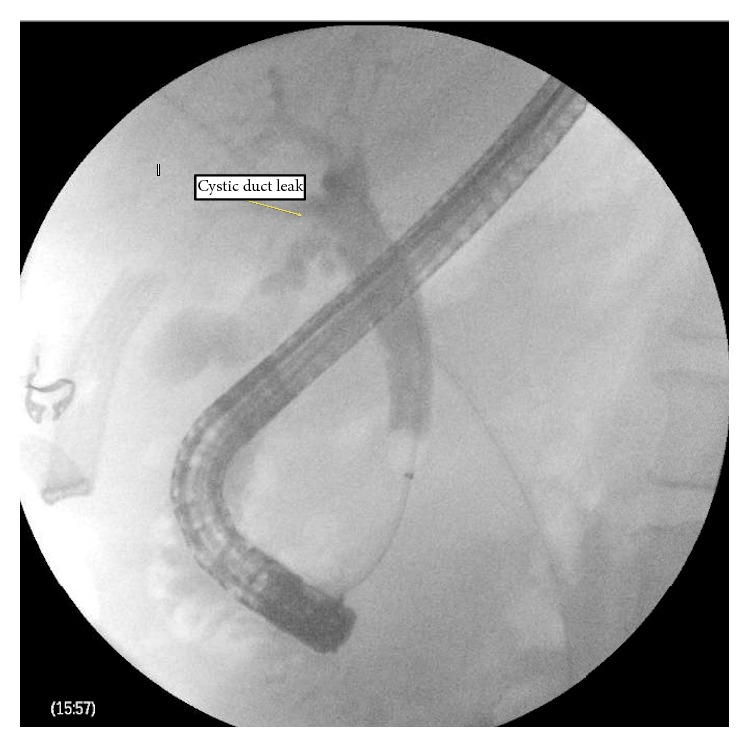
ERCP: bile leak from cystic duct.

The patient initially did well but, on the second postoperative day, a significant amount of bile was noticed in the drain. An endoscopic retrograde cholangiopancreatography (ERCP) was performed and revealed a bile leak from the cystic duct (Figure 1). Biliary sphincterotomy was performed and a 10-French × 7 cm plastic CBD stent placed. The patient did well and was discharged home on POD 5 with JP drain left in place.

**Figure 2:**
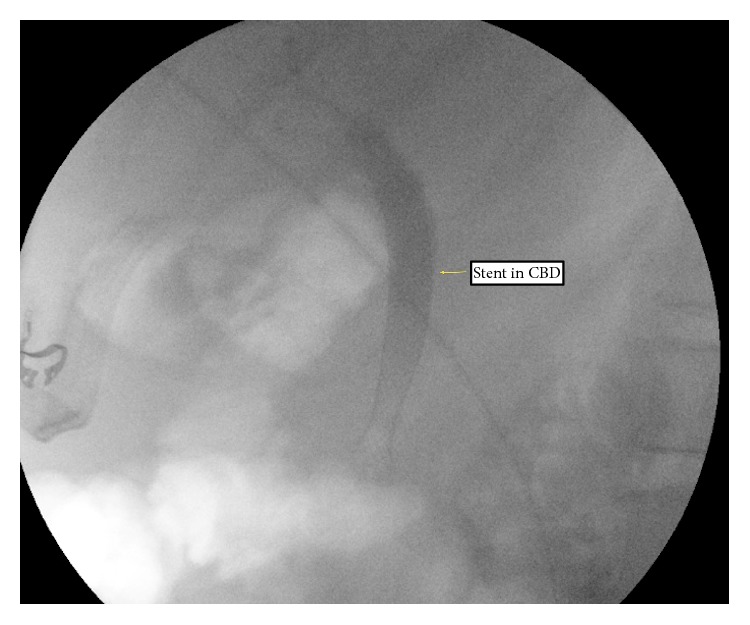
Stent in common bile duct.

During her follow-up visits, persistent leakage of bile was noted despite clinical return to baseline health status. At 8 weeks another ERCP was performed which confirmed an ongoing bile leak from the cystic duct stump. Consequently, the original stent was replaced by a fully covered temporary 10 × 60 millimeter metal CBD stent (Figure 2).

As the bile leak persisted, treatment options were discussed with gastroenterologist and interventional radiologist. Surgical option was also considered as the last resort.

**Figure 3:**
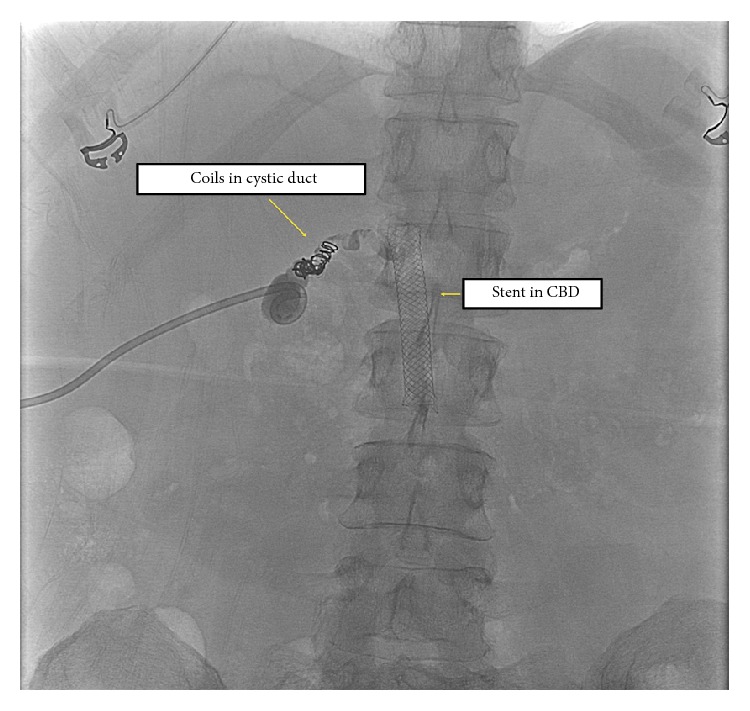
Coiling the cystic duct.

After discussion (5 weeks from last ERCP), the tract was accessed by interventional radiologist. Contrast study showed that the covered stent was not covering the origin of the cystic duct. The cystic duct was coiled with total of 5 Tornado embolization coils (Cook Medical) (6–8 mm) (Figure 3). Follow-up cholangiogram demonstrates interval decrease in patency of the cystic duct. A pigtail drain was adjacent to the cystic duct.

**Figure 4:**
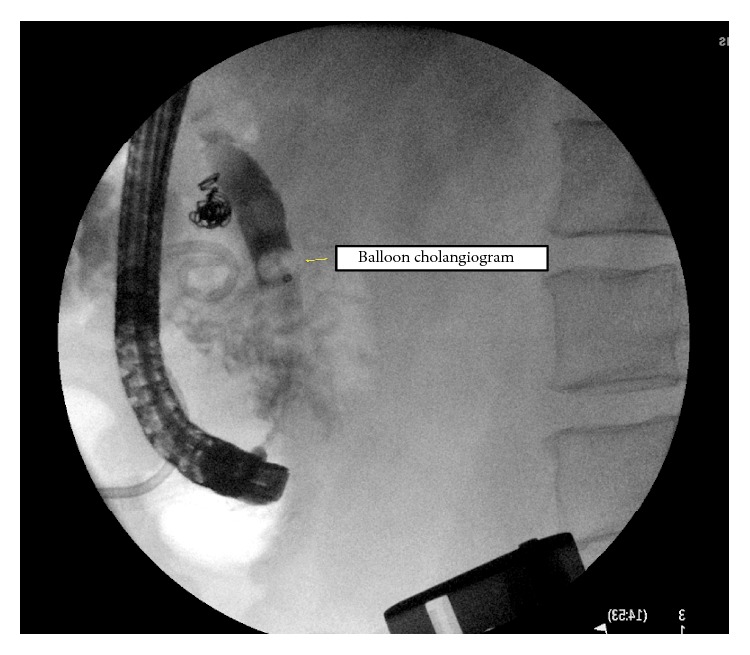
ERCP: occlusive cholangiogram with no leak.

The pigtail drain was clamped after the bile drainage stopped at 1-week follow-up. On subsequent ERCP done 2 weeks later, occlusion cholangiogram revealed no evidence of bile leak (Figure 4) and the stent and drain were removed. The patient was seen 4 months later with no further biliary complications.

## 3. Discussion

Laparoscopic cholecystectomy is one of the most commonly performed operations in the world. Bile leak from the cystic duct stump remains a significant complication of this operation [1, 2]. Bile peritonitis, subhepatic abscesses, bile duct stricture, and perihepatic inflammation leading to fibrosis have all been associated with bile leaks [3].

Endoscopic treatment at ERCP with stent and sphincterotomy is usually the first line of treatment with success rate greater than 90% [2, 5]. The median time for resolution of the leak was 3 days (range 1–39 days) [5]. Kaffes and colleagues [5] reported that stent insertion alone for postcholecystectomy bile leak is superior to sphincterotomy alone, because fewer patients required additional intervention (particularly surgery) to control the leak.

If these strategies fail, high-risk surgery (22%–37% morbidity and 3%–18% mortality) is one option [6]. Other options reported include injection of glue or coils either via endoscope or transhepatically.

Seewald et al. [7] reported their experience with endoscopic occlusion of cystic duct for bile leakage with injection of cyanoacrylate glue in 9 patients; two of them had bile leak after cholecystectomy. Other authors have also reported successful endoscopic glue injection for cystic duct leak [6]. Combination of cyanoacrylate glue and angiographic coils has also been deployed via endoscope at ERCP to resolve cystic duct leak after failed operations [8]. Percutaneous trans hepatic deployment of Hydrocoil into the cystic duct stump has been reported as well [9].

In the case presented, we used coiling of cystic duct with success to avoid operation in a patient with significant comorbidities including morbid obesity and COPD with continued smoking. To our knowledge, only another case of trans catheter cystic duct coiling has been reported in the published literature [10].

## 4. Conclusion

Trans catheter coiling of cystic duct for bile leak from cystic stump is an innovative technique, which can help avoid high-risk reoperation in patients, many of whom have significant comorbidities as in our patient. This technique can only be used in patients who have well-established drain tract.
